# Selectin Ligands Sialyl-Lewis a and Sialyl-Lewis x in Gastrointestinal Cancers

**DOI:** 10.3390/biology6010016

**Published:** 2017-02-23

**Authors:** Marco Trinchera, Adele Aronica, Fabio Dall’Olio

**Affiliations:** 1 Department of Medicine and Surgery (DMC), University of Insubria, 21100 Varese, Italy; 2 Department of Health Sciences, San Paolo Hospital, University of Milan, 20142 Milano, Italy; adele.aronica@unimi.it; 3 Department of Experimental, Diagnostic and Specialty Medicine (DIMES), University of Bologna, 40126 Bologna, Italy; fabio.dallolio@unibo.it

**Keywords:** carbohydrate antigens, glycosylation, cancer diagnosis, cancer malignancy

## Abstract

The tetrasaccharide structures Siaα2,3Galβ1,3(Fucα1,4)GlcNAc and Siaα2,3Galβ1,4(Fucα1,3)GlcNAc constitute the epitopes of the carbohydrate antigens sialyl-Lewis a (sLe^a^) and sialyl-Lewis x (sLe^x^), respectively, and are the minimal requirement for selectin binding to their counter-receptors. Interaction of sLe^x^ expressed on the cell surface of leucocytes with E-selectin on endothelial cells allows their arrest and promotes their extravasation. Similarly, the rolling of cancer cells ectopically expressing the selectin ligands on endothelial cells is potentially a crucial step favoring the metastatic process. In this review, we focus on the biosynthetic steps giving rise to selectin ligand expression in cell lines and native tissues of gastrointestinal origin, trying to understand whether and how they are deregulated in cancer. We also discuss the use of such molecules in the diagnosis of gastrointestinal cancers, particularly in light of recent data questioning the ability of colon cancers to express sLe^a^ and the possible use of circulating sLe^x^ in the early detection of pancreatic cancer. Finally, we reviewed the data dealing with the mechanisms that link selectin ligand expression in gastrointestinal cells to cancer malignancy. This promising research field seems to require additional data on native patient tissues to reach more definitive conclusions.

## 1. Introduction

The three members of the selectin family bind carbohydrate structures (and are therefore referred to as lectins) through a Ca^++^-dependent domain. They are transmembrane type I proteins with a short cytoplasmic tail and the long *N*-terminal portion containing the carbohydrate recognition domain protruding in the extracellular space. The three selectins differ in their structure and pattern of cell type expression. L-selectin is expressed on lymphocytes, monocytes and granulocytes, and is responsible for their homing through the binding of specific carbohydrate structures expressed by the high endothelial venules in lymph nodes. P-selectin is expressed by platelets and endothelial cells and is stored in membranes of α granules of platelets and in Weibel-Palade bodies of endothelial cells. It is involved in the earlier steps of the adhesion process since its mobilization to the cell surface of activated endothelial cells from the stores occurs within minutes upon the activation stimuli. E-selectin is constitutively expressed on the cell surface of venular endothelia of bone marrow and skin, while in other organs it is expressed upon stimulation by TNF-α, IL-1β, or LPS. The interaction between E-selectin and its ligands expressed on the cell surface allows leukocyte rolling, a preliminary step leading to leukocyte adhesion to endothelial cells and extravasation [1].

The minimal sugar structure required for selectin binding is constituted by an α2,3-sialylated and α1,3/4 fucosylated tetrasaccharide based on a type 1 (Galβ1,3GlcNAc) or a type 2 (Galβ1,4GlcNAc) chain. Two isomers, known as sialyl-Lewis a (sLe^a^: Siaα2,3Galβ1,3[Fucα1,4]GlcNAc) and sialyl-Lewis x (sLe^x^: Siaα2,3Galβ1,4[Fucα1,3]GlcNAc) fulfill this basic requirement. The addition of a sulfate group at the 6 position of GlcNAc generates 6-sulfo-sLe^x^, which is considered the physiologic ligand for L-selectin [2]. This epitope is carried by mucin-type *N*- or *O*-linked oligosaccharides [3].

Although selectins were originally described in the context of inflammation and immunological response, their role in cancer progression soon became evident in consideration of the role played by cancer cell adhesion to endothelia in metastasis. Concurrently, many reports were describing various glycan alterations associated with cancer, including the expression of sLe^a^ and sLe^x^ as a hallmark of the malignant phenotype [4]. Interestingly, sLe^a^ is the epitope of the CA19.9 antigen, one of the most widely used tumor markers in the clinical management of pancreatic and other gastrointestinal cancers, as well as for research purposes. Recently, a role of circulating sLe^x^ has been proposed as a marker for monitoring the metastatic progression of breast cancer [5] and has been associated with tumor invasion by a meta-analysis study [6].

In the present article, we review recent data on the biosynthesis and role of selectin ligands in gastrointestinal cancers. In particular, we will discuss how the mechanisms operating in the immunological response and inflammation are recapitulated in cancer cells, and their significance in gastrointestinal cancer patients. We will also highlight the still controversial points requiring further experimental studies.

## 2. Biosynthesis of sLe^x^ and sLe^a^

**Figure 1:**
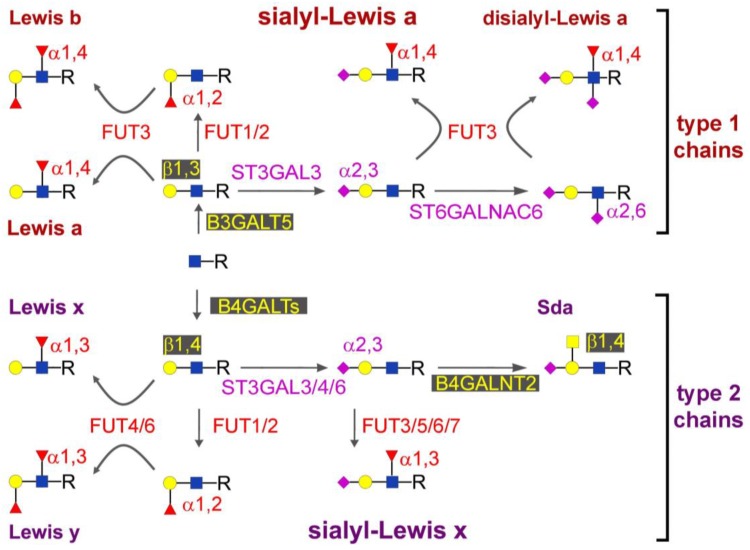
Structure and biosynthesis of Lewis antigens. Monosaccharides are depicted. Blue square: *N*-acetylglucosamine (GlcNAc); yellow square: *N*-acetylgalactosamine (GalNAc); yellow circle: galactose (Gal); red triangle: fucose (Fuc); pink diamond: sialic acid (Sia). Anomers, linkage positions, and enzymes involved in the reactions are indicated. All enzymes known to be able to perform a reaction are listed, note that only some of them are proven to be expressed in gastrointestinal tissues, as detailed in the text.

The biosynthesis process of sLe^a^/sLe^x^ (Figure 1) has been known since the nineties [7]. However, the actual contribution of the different α2,3 sialyltransferases and α1,3/4 fucosyltransferases to the biosynthesis of selectin ligands in different healthy and cancer gastrointestinal tissues is far from established. This is due to the complex tissue-specific pattern of expression of these enzymes and the difficult use of the mouse model [7,8]. Moreover, the genetic manipulation of cell lines by cDNA transfection or gene silencing [9,10,11,12,13] provides mainly indications on the potential role of a glycosyltransferase in elaborating a sugar epitope in an experimental system, rather than on its real role in normal or cancer tissues. For example, in pancreatic cancer cell lines, both ST3GAL3 and ST3GAL4 are able to induce sLe^x^ biosynthesis [10,14], although the over-expression of sialyl-Lewis (sLe) antigens in surgical specimens is not correlated with either gene [15]. In normal and cancer colonic tissues, the α2,3-sialylation step of sLe^x^ biosynthesis appears to be mediated by ST3GAL4, owing to the very low expression of ST3GAL3 [16], while the α1,3-fucosylation step is mediated mainly by FUT6 [17]. However, the overexpression of sLe^x^ in colon cancer tissues cannot be explained by the up-regulation of any single glycosyltransferase [16,18,19]. In a recent review [20], we have proposed that the right question to answer is not “why are sLe^x^ levels high in colon cancer?” but rather “why are sLe^x^ levels low in the normal colon?” Competition between FUT6 and enzymes synthesizing alternative structures, such as Siaα2,3(GalNAcβ1,4)Galβ1,3/4GlcNAc (the Sd^a^ antigen) [21,22] or the sialyl 6-sulfo-Lewis x antigen [23] has been proposed. The Sd^a^ antigen is the product of β1,4 *N*-acetylgalactosaminyltransferase-II (B4GALNT2) [24,25]. Both the Sd^a^ antigen and B4GALNT2 are highly expressed in normal colon and down-regulated in colon cancer [26,27]. The expression of B4GALNT2 induces down-regulation of sLe^x^ in colonic and gastric cancer cell line [21,22] and probably also in normal colonic tissues.

In fact, while in colon cancer tissues (where B4GALNT2 levels are low) sLe^x^ levels correlate with *FUT6* expression [17], in normal colon (where B4GALNT2 is high), they correlate with the FUT6/B4GALNT2 ratio [28].

The branching point and the possible competition between the biosynthesis of type 1 and 2 chains, (and consequently between sLe^a^ and sLe^x^) is mainly controlled by the relative levels of expression of β1,3- and β1,4-galactosyltransferases. Seven β1,4-galactosyltransferase enzymes (B4GALT1–7) are known in humans [29]. B4GALT1, the first cloned glycosyltransfease and one of the most deeply investigated, is the one forming with lactalbumin the lactose synthase complex [30]. Association of B4GALT1 with sLe^x^ expression has been reported in cancer [31] but little data on the other members of the family are available. Up-regulation of type 2 chains [32], of B4GALT1 [33], and B4GALT4 [34] has been reported in colon cancer and is associated with malignancy.

Four β1,3-galactosyltransferases (B3GALTs) have been described in humans: B3GALT1–2 and B3GALT4–5. Much less data are available on B3GALTs than on B4GALTs. Sometimes they are limited to a single report, as in the case of B3GALT2 [35], whose function is totally unknown. The role of B3GALT5 in gastrointestinal tissues is rather well defined. In vivo and in cell models its expression is closely associated with that of Lewis type 1 antigens, including sLe^a^ [36,37]. B3GALT5 acts on *N*- and *O*-linked chains of glycoproteins, on glycolipids, and on soluble type 1 chain oligosaccharides [37,38]. In glycolipids, it is specifically responsible for globo-series elongation [39]. In Chinese hamster ovary (CHO) cells, it competes with B4GALTs, blocking poly-*N*-acetyllactosamine chain elongation and sLe^x^ synthesis [37], while its silencing in the pancreatic cancer cell line BxPC-3 reduces sLe^a^ expression and increases sLe^x^ secretion [38]. B3GALT5 is strongly expressed in the normal colonic mucosa, resulting in Lewis a and, to a much lower extent, in sLe^a^ expression. Conversely, the B3GALT5 transcripts are down-regulated in colon cancer, where type 1 Lewis antigens are scarcely or not detectable [37,40,41]. In normal and pancreatic cancer tissues the amount of B3GALT5 mRNA is similar, and lower than in the colon mucosa. In both cases, type 1 chain Lewis antigens are well detectable, but only in the glandular ducts or in the ductal-like structures present in the relatively well differentiated adenocarcinomas [40]. In CHO cells, B3GALT1 appeared to be able to synthesize sLe^a^ [37] and it was recently proposed as the enzyme responsible for sLe^a^ synthesis in other tissues, such as the prostate [42]. Notably, in cell lines derived from gastrointestinal cancers, the expression of sLe^a^ and/or sLe^x^ is rather infrequent. The cell lines COLO-205, SW-1116 and LS-174T express sLe^x^, and the first two surprisingly express also high amounts of sLe^a^. This finding apparently contradicts the sLe^x^/sLe^a^ competition revealed by the studies in CHO and BxPC3 cells above reported, and suggests that multiple variables affect the relative expression of such antigens in vivo.

Another important biosynthetic step of selectin ligand biosynthesis is represented by the addition of the GlcNAc residue preceding the galactosyl residues. The GlcNAc transferases involved differ depending on the nature of the carbohydrate chain. On *N*-linked chains, the elongation of the polylactosaminic chains bearing sLe^x^ occurs mainly in the β-1,6 branching, whose biosynthesis is controlled by the metastasis-associated enzyme GnT5 [43]. On the *O*-linked chains of glycoproteins, sLe^x^/sLe^a^ expression is strongly dependent on Core 2 GlcNAc transferase (C2GnT) [44], while in glycolipids a pivotal role in their biosynthesis appears to be played by a β1,3GlcNAc transferase which synthesizes a common precursor of both type 1 and 2 chain Lewis structures [45]. Interestingly, this enzyme is activated by *Helicobacter pylori* infection, leading in stomach cells to increased expression of sLe^x^, which is a ligand for *H. pylori* adhesin SabA [46].

## 3. Regulation of sLe^a^ and sLe^x^

Unfortunately, the knowledge of glycosyltransferase gene regulations and its relation to cancer is still preliminary. Epigenetic control is supposed to be relevant in many cases [47,48], while other emerging mechanisms controlling gene expression, such as the presence of regulatory RNA sequence and or RNA binding proteins [49], are, as yet, unexplored. At present, data are available about promoter sequences and potential regulatory elements operating in various tissues [50,51,52,53,54,55,56,57], while those actually involved in gastrointestinal cancer are described in very few cases [43,58,59,60,61]. This is a very promising field of research since a bidirectional relationship may exist between the expression of sLe antigens and the mechanisms controlling cell growth. For instance, the expression of the metastasis-suppressive gene *Nm23-H1* in a human hepatocarcinoma cell line induces down-regulation of both FUTs and ST3GALs, resulting in reduced sLe^x^ expression [62]. On the other hand, sLe antigens expressed on the TGF-β receptor enhance colon cancer cell migration through promotion of the epithelial to mesenchymal transition [63], which instead was found associated to the activity of the *ST6GAL1* gene in other cancer cell lines [64].

It is well known that oncogenes are able to activate glycosyltransferases involved in the biosynthesis of cancer-related carbohydrate structures. Good examples are provided by the *MGAT5* gene which is up-regulated by src [65], ErbB2 [66], v-sis [67] and Ras [68,69] oncogenes, through Ets-1 transcription factor [70,71] and by sialyltransferase ST6GAL1, which is regulated by both *N*-ras and *H*-ras through RalGEF signaling [72,73,74,75,76]. Also, the biosynthesis of Lewis type structures is affected by oncogenes. In gastric cancer cells, the forced expression of ST3GAL4 led to increased invasive potential through the biosynthesis of sLe^x^, which, in turn, activates the c-Met pathway [77]. In addition, the expression of the Lewis y antigen, induced by the forced expression of FUT4 [78,79] or of FUT2 [80], led to increased cell growth through activation of a signal transduction pathway generated at the cell membrane level. Colon cancer cells induced to epithelial to mesenchymal transition by EGF and/or bFGF, displayed increased sLe^a^/sLe^x^ expression because of up-regulation of ST3GAL1, -3 and -4 and of FUT3, with a concomitant decrease of FUT2. These effects were dependent on c-myc expression [81]. Altogether, these data indicate that both sialylated (sLe^x^/sLe^a^) and non-sialylated (Lewis y) Lewis type antigens, when expressed on cell membrane receptors, can activate an “outside-in” (from the cell membrane to the nucleus) signal transduction pathways leading to increased growth and malignancy. On the other hand, expression of activated oncogenes leads to overexpression of Lewis-type antigens through glycosyltransferase stimulation, generating an “inside-out” (from the nucleus to the cell membrane) flow of information. It can be hypothesized that these two opposite flows of information generate a loop self-fueling invasive cell growth.

Some reports also suggested that sLe expression is regulated by mechanisms alternative to glycosyltransferase regulation. They include the cancer-associated expression of nucleotide-donor transporters [82,83,84] and hypoxia [85], sometimes working in tandem [83]. These data were not further developed and need to be evaluated on the light of recent papers dealing with the derangement of fucosylation occurring in cancer. A general loss of the fucosylation potential has been reported in fact in up to 13% of colorectal cancers due to mutations of an enzyme involved in the biosynthesis of GDP-Fuc [86,87], the mandatory donor of fucose in all fucosyltransferase-catalyzed reactions. Such mutation and the consequent loss of fucosylation was very recently reported to enhance inflammation and tumors in the mouse [88]. On the other side, a general increase of fucosylation was also reported in colorectal cancer, as suggested by the binding of *Aleuria aurantia* lectin [89], that preferentially recognizes fucose α1,2/3/6 linked to lactosamine units [90]. Increased fucosylation levels were also reported in cancer stem cells of the pancreas and associated to chemoresistance in pancreatic cell lines [91].

## 4. Plasma Carriers of sLe^x^ or sLe^a^

Owing to the role of sLe antigens in cancer diagnosis, the nature of their carrier molecules in plasma is of crucial importance. In fact, the global level of expression of these antigens in plasma does not always provide the sufficient sensitivity and/or specificity to discriminate cancer from non-malignant conditions, nor to follow disease progression, while the presence of sLe epitopes on specific glycoproteins can provide the necessary sensitivity and/or specificity. For example, in pancreatic cancer (Table 1), the CA19.9 antigen has been reported to be carried by MUC1, MUC5AC and MUC16 [92,93], and also by apolipoprotein B-100, kininogen and apolipoprotein E [94]. However, while on MUC16 the up-regulation of CA19.9 parallels that of the protein carrier (indicating that no real change of glycosylation occurred), on MUC1 and MUC5A the elevation of CA19.9 was not due to the increased expression of the protein carrier [93]. The protein carrier may reflect the disease state.

In fact, MUC16 is the most prevalent CA19.9 carrier in pancreatitis, while MUC5AC and MUC1 are the preferential carriers in cancer. Acute phase proteins are carriers of sLe^x^ in pancreatic diseases [95]. In particular, increased sLe^x^ expression on α1-acid glycoprotein occurs in both cancer and chronic pancreatitis, while increased expression on haptoglobin, fetuin, antitrypsin and transferrin is associated only with chronic pancreatitis, not with pancreatic cancer [95]. Ceruloplasmin is a carrier of sLe^x^ in pancreatic diseases; an increase of the sLe^x^/ceruloplasmin ratio is present in pancreatic cancer [96]. In plasma of pancreatic cancer patients, CA19.9 is carried also by glycolipids associated with the bile globular membrane, a membrane vesicle secreted by normal bile [97]. In some colorectal cancer patients, sLe^x^ is carried by α1-acid glycoprotein in both tissues and plasma [98]. By using high density antibody arrays, a variety of glycoproteins expressing sLe^a^ and/or sLe^x^ were identified in plasma of colon cancer patients [99]. SPARC, coagulation factor V, neuropeptide Y, complement C2 and α2-macroglobulin were among the glycoproteins more strongly expressing sLe^a^. These glycoproteins (and many others) also expressed sLe^x^. In pancreatic and colon cancer cell lines, MUC1 is a carrier of various Lewis type antigens, including sLe^a^ and sLe^x^ [100]. In gastric cancer, an increased expression of sLe^x^, carried by acute response proteins released by the liver, is probably dependent on the systemic inflammatory status associated with cancer [101].

Interestingly, the fucosylation pattern of some serum proteins is specifically affected in gastrointestinal cancer patients. For example, pancreatic ductal adenocarcinomas are associated with increased α1,3fucosylation of α1-acid glycoprotein, which is of hepatic origin [102], while colorectal cancers are associated with altered core-fucosylation of IgG glycans, which are produced by plasma cells [103]. In particular, the core-fucosylation was increased in non-sialylated IgG glycans and decreased in those sialylated. Since the glycosylation of IgG glycans is strongly dependent on the inflammatory status (reviewed in [104]), it is possible that the cancer-associated systemic inflammation affects the glycosylation machinery of distant tissues. Pro-inflammatory cytokines have been reported to regulate the expression of glycosyltransferases involved in the biosynthesis of disease-associated glycans. In particular, TNF was found able to up-regulate ST3GAL4 in human lung increasing sLe^x^ expression [105]. It is worth recalling that sLe antigens can be carried by a glycosphingolipid backbone, forming gangliosides. sLe gangliosides potentially circulate in the bloodstream in the form of membrane vescicles [97], and are actually effective selectin ligands [106,107]. In colon cancer cells, sLe^a^ ganglioside was found able to bind E-selectin and promote cell adhesion upon removal of mucin carrying sLe^a^ [108], suggesting a potential cooperation between the various carrier structures.

## 5. Role in Diagnosis

**Figure 2:**
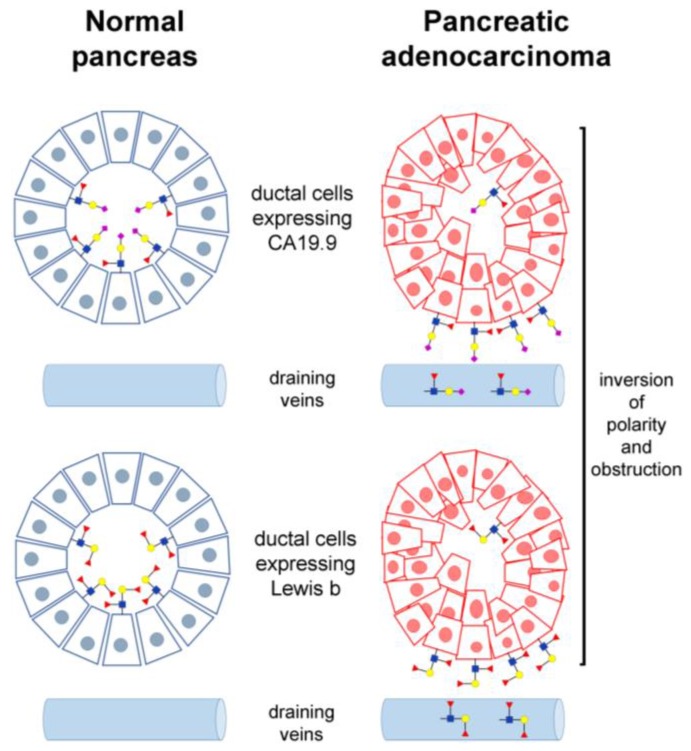
Proposed model of the elevation of circulating CA19.9 in pancreatic cancer patients. The inversion of polarity occurring in malignant ductal cells [125] together with the obstruction of the cancerous ducts determine reabsorption by the blood of the molecules normally secreted by the organ [121,124]. According to recent data [40], the expression of type 1 chain Lewis antigens in the pancreas is regulated by the individual glycosyltransferase pattern, which is not deregulated in cancer. Consequently, serum CA19.9 levels increase only in those patients actually expressing the antigen in their normal ducts. Non-malignant pancreatic diseases causing obstruction, such as chronic pancreatitis, are expected to determine partially overlapping elevation of circulating antigens. Sugars are depicted as in Figure 1.

Due to the general derangement of the glycosylation machinery associated with cancer [109], many carbohydrate structures are potential markers for early diagnosis of gastrointestinal cancers. However, sLe^a^ remains the most consolidated and used carbohydrate tumor marker. As the epitope of CA19.9 antigen, it represents the target of the monoclonal antibody 1116-NS-19-9 developed 30 years ago immunizing mice with the colon cancer cell line SW-1116. At present, the antigen is determined for multiple purposes in the serum of patients suffering various gastrointestinal diseases, leading to an enormous number of clinical determinations whose rationale is questioned [110,111]. According to the American Society of Clinical Oncology [112] and the European Group on Tumor Markers [113], determination of CA19.9 is recommended only for the follow-up, surveillance, and response to therapy of pancreatic cancer. Taking into account the prevalence of pancreatic cancer, the expected number of CA19.9 determinations was calculated to be 10-fold lower than the number of tests actually performed in a western country [111].This is probably due to the assumption that serum CA19.9 values may help the differential diagnosis of several gastrointestinal diseases, including cancers arising not only in the pancreas [114,115,116], but in the colon [117], stomach [118,119], and bile ducts [120]. In this regard, it is important to recall that the synthesis, expression, and secretion of CA19.9 was demonstrated in many healthy tissues, including the pancreas [121] and the bile ducts [122]. In fact, both the pancreatic juice and the bile physiologically contain relevant amounts of the antigen. The question is why blood CA19.9 increases in some patients but not in others, and why elevated levels of serum CA19.9 are found in cancer patients and in patients affected by non-malignant diseases as well. The highest CA19.9 serum values are reported in severely jaundiced patients [123], while the elevation in chronic pancreatitis is lower and overlapping that of pancreatic cancer patients [124]. To explain these observations, several years ago an hypothesis was formulated proposing that in pancreatic cancer the increased serum CA19.9 levels were due to the inversion of polarity of transformed ductal cells [125] and to the obstruction of the ducts, which in turn give rise to the abundant reabsorption of the antigen in the blood [124]. On the other hand, immunohistochemical detection suggested an abundant and rather specific expression of CA19.9 in pancreas and colon cancer, and not in the surrounding normal tissue [126,127,128]. Normal colon was rather considered to express the structurally related antigens Lewis a and Lewis b [129]. These observations were further consolidated by the discovery of disialyl-Lewis a in healthy colon mucosa, due to the activity of the sialyltranferase ST6GALNAC6 (Figure 1). It was proposed that sLe^a^ expression in colon cancer was due the loss of disialyl-Lewis a because of the down-regulation of ST6GALNAC1 [130]. However, differential expression of disialyl-Lewis a is not known in organs other than the colon, and colon cancer is associated with a strong down-regulation of B3GALT5 [37,41,131], the key enzyme in the synthesis of all Lewis type 1 antigens, including sLe^a^ (Figure 1). Coherently, CA19.9 was scarcely detected in colon cancer by dot-blotting [40,132] or western blotting [16]. This apparent contradiction was recently explained by the finding that using the 1116-NS-19-9 anti-CA19.9 antibody by immunohistochemistry generates artifacts [133]. In contrast, the use of the same antibody by immunofluorescence correctly confirmed the absence of CA19.9 in colon cancer, suggesting that the antigen circulating in such patients may originate outside the colon, i.e. from the bile [40]. Immunofluorescence also showed that in normal pancreas, CA19.9 expression is restricted to the ducts, and in cancer it is restricted to ductal like structures, while the bulk of the malignant cells lacks expression. Altogether, these data suggest a re-evaluation of the hypothesis, based on the obstruction of the ducts and the reverse of polarity, which provides a more satisfactory explanation for serum CA19.9 elevation. This hypothesis predicts that other carbohydrate antigens synthesized and secreted by pancreatic ductal cells may undergo reabsorption in the blood due to obstruction and reverse of polarity occurring in cancer (Figure 2). Recently, Lewis b was actually found expressed in a pancreatic cancer patient lacking CA19.9 [40], suggesting that such antigen may turn useful for monitoring CA19.9-negative patients.

In the future, it would be advisable to detect all Lewis antigens in tissue sections of surgical specimens by immunofluorescence and compare the results with the clinical records of the patients, including their serum CA19.9 levels. In fact, a relevant issue to be addressed concerns the potential expression of sLe^x^ in conjunction or alternative to CA19.9. Recently, it was proposed that CA19.9-negative cancer patients may be monitored through circulating sLe^x^ [134], and the expression of sLe^x^ in distinct glycans appears to be independent of that of sLe^a^, potentially improving the diagnostic accuracy over CA19.9 [135]. The expression of sLe^x^ on plasma protein seems to be actually associated with gastrointestinal cancers, as above reported [96].

## 6. Role in Malignancy

sLe antigens may promote metastasis by forming emboli of cancer cells and platelets, which favor their arrest on endothelia [136,137,138,139]. In colon cancer patients, increased expression of sLe^x^ and sLe^a^ correlated with metastasis and poor survival [140,141,142,143]. Down-regulation of sLe expression by knock-down of key glycosyltransferases in cancer cell lines resulted in reduced selectin binding and reduced metastatic ability [11,12,144], while forced expression of sLe antigens by gene transfer increased adhesion to selectins in vitro and metastatic ability in vivo [10]. The role of selectins in the metastatic process was confirmed by the finding that the formation of experimental pulmonary metastases could be inhibited by the use of peptides mimicking sLe^a^ and was inhibited in E-selectin- knock-out mice [145]. Owing to the fact that mesothelial cells express, like endothelial cells, E- and P-selectins, the two adhesion molecules are crucial also for peritoneal spread of pancreatic carcinoma [146].

Beside adhesion, sLe antigens influence also angiogenesis and immune recognition of cancer cells. The role of sLe^x^ in angiogenesis is indicated by the observation that the formation of tube-like networks of endothelial cells induced by the co-culture with cancer cells can be inhibited by antibodies against sLe^x^ [147]. Moreover, the ability of hepatocarcinoma cells to promote angiogenesis was inhibited by blocking sLe^x^ biosynthesis [148]. On the other hand, forced expression of sLe^a^ and type 1 Lewis antigens in a colon cancer cell line by transfection with B3GALT5 also resulted in increased angiogenesis and tumor growth [133]. Altogether, these results suggest that angiogenesis is stimulated by sLe antigens, regardless of the underlying carbohydrate structure (sLe^a^ or sLe^x^). Cells expressing high levels of sLe^x^ are a better target of natural killer cells and thus less metastatic than those expressing moderate levels of the antigen [149,150].

Many of the experimental models reported above utilized subsets of cancer cell lines or engineered cell clones differing in the expression of the terminal sLe epitopes, which are seeded in contact with endothelial cells in vitro, or inoculated in nude mice to form xenografts. Although their presence is the basic requirement for selectin binding, the mere over-expression of sLe^a^ and/or sLe^x^ does not necessarily imply an increase of the adhesion properties of the cells. Conversely, a shift from the expression of a particular carrier molecule to another has the potential to alter more deeply the adhesion properties of the cells. At present, the actual expression of sLe antigens in native gastrointestinal cancers is not adequately known because of the little available data [151,152], potentially affected by artifacts [127,128,141]. Particularly, in colon cancer evidence of the expression of genuine CA19.9 is missing, and elevation of the circulating antigen is reported mainly in advanced stages of the disease [153,154]. In none of cases reported in vivo was any information available about the nature of the carrier molecules.

## 7. Concluding Remarks

A large number of clinical and experimental data is now available through many papers dealing with the relationship between the expression of sLe antigens and the phenotype of various cancer types, including those of gastrointestinal origin. The biosynthesis of sLe antigens is mainly controlled through the regulation of several glycosyltransferase genes, which has been elucidated only for few of them. Consequently, it is not yet possible to link sLe expression to the mechanisms controlling cell growth. It is well established that sLe expression has the potential to deeply influence the phenotype of cancer cells by modulating the adhesion properties, the angiogenic ability, and the immune recognition. However, the actual and reciprocal expression of sLe^a^ or sLe^x^ in native gastrointestinal cancers is still questioned and needs to be unambiguously confirmed in the near future. In particular, recent data indicated that the detection by histochemistry of sLe^a^/CA19.9 by the NS-1116-19-9 antibody provided artifacts, suggesting re-evaluation of Lewis antigen expression by immunofluorescence. Moreover, the nature of the carrier molecules and their ability to constitute the proper scaffold promoting cell adhesion in vivo requires further attention. In particular, the peculiar role of gangliosides as selectin ligands deserves dedicated experiments. The presence of these antigens has been identified on a variety of protein and non-protein carriers in the plasma. Selectin ligands associated with specific carrier molecules represent a class of novel markers potentially useful in early diagnosis, as they are able to discriminate neoplastic and non-neoplastic diseases. At present, the simple detection of sLe antigens fails in targeting such clinical aims. In several cases, these antigens are carried by molecules that presumably have not been synthesized by the cancer cells, indicating an indirect mechanism for their overexpression. The proposed biliary origin of circulating CA19.9 in colon cancer patients [40,133] needs further investigation but provides a paradigm of the indirect mechanisms of production of carbohydrate markers circulating in some cancer patients.

## Figures and Tables

**Table 1:** Plasma protein-carrying sialyl-Lewis (sLe) antigens in pancreatic diseases. sLe^a^: sialyl-Lewis a; sLe^x^: sialyl-Lewis x.

Selectin Ligand	Carrier Molecule	Disease Involved	Reference
sLe^a^	MUC1	cancer > chronic pancreatitis	[92,93]
sLe^a^	MUC5AC	cancer > chronic pancreatitis	[92,93]
sLe^a^	MUC16	chronic pancreatitis > cancer	[92,93]
sLe^a^	Apo-B-100	cancer = chronic pancreatitis	[94]
sLe^a^	Apo-E	cancer = chronic pancreatitis	[94]
sLe^a^	Kininogen	cancer = chronic pancreatitis	[94]
sLe^x^	α1-acid glycoprotein	cancer > chronic pancreatitis	[95]
sLe^x^	Ceruloplasmin	cancer > chronic pancreatitis	[95]
sLe^x^	Haptoglobin	chronic pancreatitis > cancer	[95]
sLe^x^	Fetuin	chronic pancreatitis > cancer	[95]
sLe^x^	Antitrypsin	chronic pancreatitis > cancer	[95]
sLe^x^	Transferrin	chronic pancreatitis > cancer	[95]
